# FUSE: data-driven functional segmentation of DNA methylation data

**DOI:** 10.1093/bioinformatics/btag201

**Published:** 2026-04-29

**Authors:** Susanna Holmström, Antti Häkkinen, Kari Lavikka, Giovanni Marchi, Sampsa Hautaniemi, Alexandra Lahtinen

**Affiliations:** Research Program in Systems Oncology, Research Programs Unit, Faculty of Medicine, University of Helsinki, Helsinki, 00014, Finland; Computational Health Informatics Program, Boston Children’s Hospital, Harvard Medical School, Boston, MA, 02215, United States; Research Program in Systems Oncology, Research Programs Unit, Faculty of Medicine, University of Helsinki, Helsinki, 00014, Finland; Research Program in Systems Oncology, Research Programs Unit, Faculty of Medicine, University of Helsinki, Helsinki, 00014, Finland; Research Program in Systems Oncology, Research Programs Unit, Faculty of Medicine, University of Helsinki, Helsinki, 00014, Finland; Research Program in Systems Oncology, Research Programs Unit, Faculty of Medicine, University of Helsinki, Helsinki, 00014, Finland

## Abstract

**Summary:**

DNA methylation (DNAm) of neighbouring CpG sites is highly correlated, making DNAm function in terms of blocks. DNAm patterns and functionality are linked to both chromatin structure of DNA and gene regulation. Defining biologically meaningful DNA methylation blocks from whole-genome bisulfite sequencing (WGBS) data remains challenging, as most existing methods rely on fixed genomic windows rather than the observed methylation pattern. We present FUSE, a data-driven segmentation method that captures intrinsic methylation segments directly from WGBS data by jointly analyzing multiple samples. FUSE identifies spatially homogeneous methylation blocks shared across the input cohort while allowing different methylation states across samples. Applied to 61 WGBS samples from the ENCODE database, FUSE identified segments which overlap significantly with promoters, enhancers, and repetitive elements. FUSE was able to recover the true segment breakpoints in synthetic data with high sensitivity under increased levels of noise. As such, FUSE facilitates post hoc methylation analyses by aggregating coherent CpG sites into candidate segments for downstream differential methylation testing or other comparative studies.

**Availability and implementation:**

FUSE is implemented as an R-package *methFuse*, available at https://github.com/holmsusa/methFuse and https://cran.r-project.org/package=methFuse. A GenomeSpy visualization of the data is available at https://csbi.ltdk.helsinki.fi/p/fuse_encode_gs/

## Introduction

DNA methylation (DNAm) is an epigenetic mechanism involved in gene expression, cell differentiation, genomic imprinting, and repression of retroviral elements (Smith et al. 2025). In animal genomes, DNAm predominantly occurs as 5-methylcytosine at CpG dinucleotides (Feng et al. 2010). Aberrant methylation patterns are strongly linked to cancer progression and drug resistance (Song et al. 2025).

CpG methylation levels are shaped by local CpG density and nearby methylation states (Haerter et al. 2014). Adjacent CpG sites show strong correlations in methylation levels, particularly in CpG-dense regions, and the DNAm pattern is stably maintained through cell division (Affinito et al. 2020). Thus, methylation acts at the level of blocks rather than individual sites, making segment-level analyses more informative than single-CpG approaches.

The most prominent block-like structures are CpG islands (CGIs), commonly found in promoters of housekeeping genes (Gardiner-Garden and Frommer 1987) and linked to chromatin features such as H3K4me3 (Thomson et al. 2010). Promoters and enhancers are regulated by chromatin structure and spatial DNA conformation (Whalen et al. 2016), as well as by DNA methylation, which therefore encodes spatial and regulatory information.

Existing segment-based DNAm methods focus largely on differential methylation and cell-type specific marks, but a method to extract blocks capturing the global intrinsic patterns is lacking. Instead, studies often rely on fixed windows or predefined annotations as proxies for functional regions. To address this gap, we introduce FUSE, a data-driven segmentation method that extracts intrinsic methylation blocks from whole-genome bisulfite sequencing (WGBS) data by jointly analyzing multiple samples.

FUSE identifies blocks of CpG sites that capture spatially coherent methylation patterns while allowing variation in methylation states across cell types. The resulting segmentation provides a cohort-level representation of methylation domains that can be explored at multiple resolutions and serves as a framework for downstream analyses, such as segment-level differential methylation testing or other comparative studies across samples. We show that coherent FUSE segments obtained using Bayesian Information Criterion (BIC)-based model selection show expected significant enrichment for enhancers, promoters, H3K4me3, and repetitive elements.

## Implementation

### Modeling methylation

Bisulfite treatment of DNA converts unmethylated cytosines to thymines, while methylated ones are preserved. The number of methylated counts at a CpG site can be modeled using a binomial distribution, where the read depth is the number of independent trials. Let $T\in N^{m\times n}$ and $C\in N^{m\times n}$ denote matrices of unmethylated and methylated read counts, respectively, with *m* CpG sites and *n* samples.

For each site i=1,…,m and sample j=1,…,n, we define the methylation counts $X_{ij}\in N$ as


(1) $$X_{ij}∼\text{Binomial}(t_{ij}+c_{ij},p_{ij}),$$

where $t_{ij}$ and $c_{ij}$ are the unmethylated and methylated read counts, respectively, and $p_{ij}\in [0,1]$ is the methylation rate of site *i* in sample *j*.

The maximum likelihood estimate (MLE) of the methylation rate for site *i* in sample *j* is


(2) $${\hat{p}}_{ij}=\frac{c_{ij}}{t_{ij}+c_{ij}},$$

which corresponds to the site’s β-value. For a subset of CpG sites S⊆{1,…,m}, the aggregated methylation rate for sample *j* is


(3) $${\hat{p}}_{S,j}=\frac{\sum_{i\in S}c_{ij}}{\sum_{i\in S}\left(t_{ij}+c_{ij}\right)}.$$

The binomial likelihood for the observed counts in the subset S of sites is


(4) $$L_{S}(\{p_{S,j}\})=\prod_{i\in S}\prod_{j=1}^{n}\left(\begin{array}{c} t_{ij}+c_{ij}\\ c_{ij} \end{array}\right)p_{S,j}^{c_{ij}}{\left(1-p_{S,j}\right)}^{t_{ij}}.$$

Evaluated at the MLE ${\hat{p}}_{S,j}$, the log-likelihood becomes


(5) $$\text{log}\;{\hat{L}}_{S}=\sum_{i\in S}\sum_{j=1}^{n}\left[c_{ij}\;\text{log}\;{\hat{p}}_{S,j}+t_{ij}\;\text{log}(1-{\hat{p}}_{S,j})\right].$$

### Hierarchical clustering

FUSE employs agglomerative hierarchical clustering to obtain a multiresolution segmentation of the genome. While hierarchical clustering does not guarantee a globally optimal solution, it avoids imposing directional transition assumptions or specific parametric constraints on segment lengths, as in commonly used hidden Markov models (HMMs) for genomic segmentation and yields segmentations at multiple resolutions in a computationally efficient manner.

**Figure 1:**
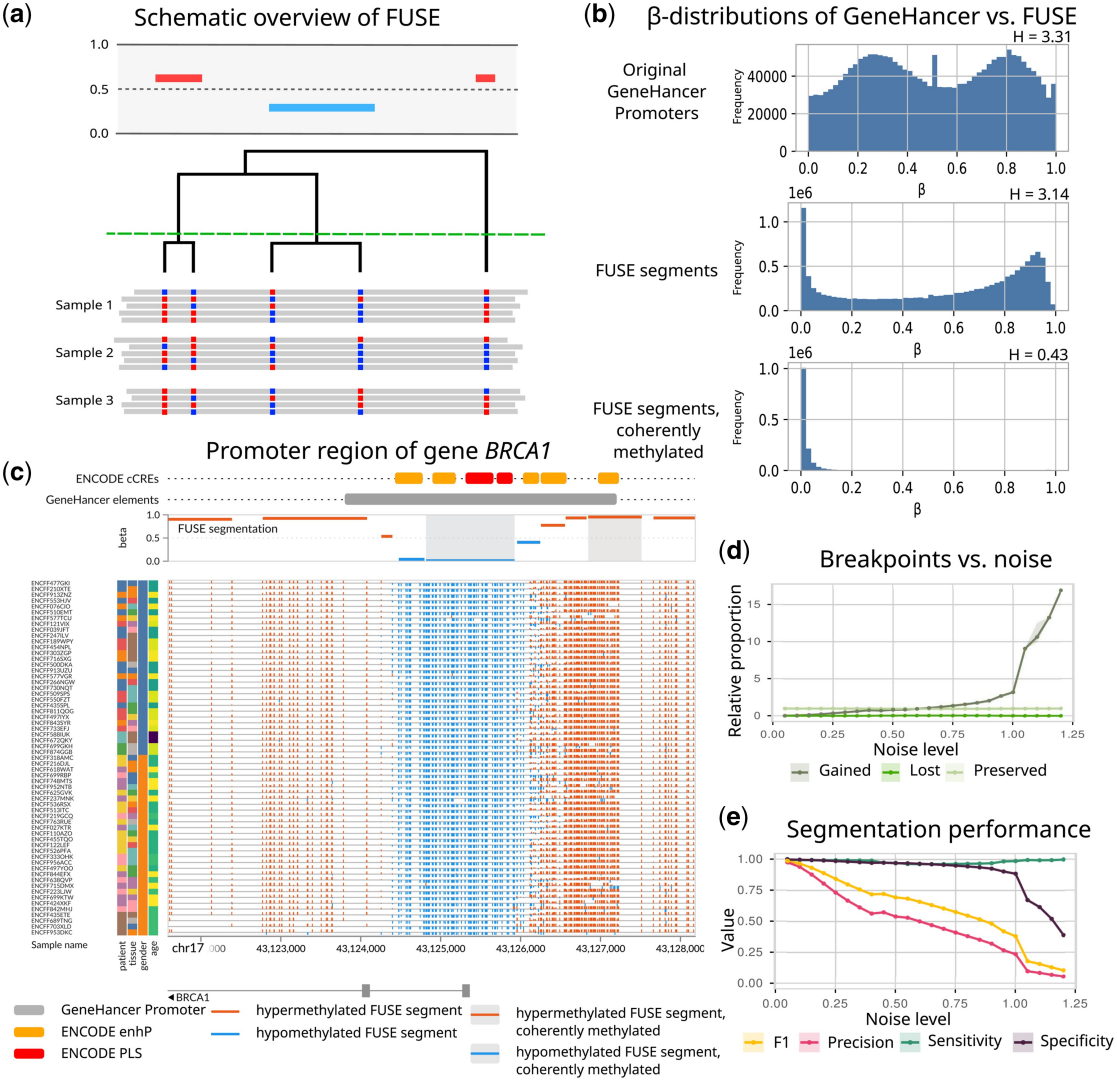
(a) Schematic overview of the FUSE-clustering. CpG sites are clustered hierarchically, and cutting the tree results in segments. (b and c) Results of the whole-genome segmentation of the ENCODE data set described in the text. (b) Comparison of beta distributions in promoter regions from GeneHancer. The upper plot shows the promoters as they are annotated, the middle plot shows all FUSE-segments overlapping with these promoters, and the lower plot shows all coherently methylated FUSE segments overlapping with these promoters. Entropy (H) decreases from top down, indicating a reduction in uncertainty within the data. (c) Snapshot of a GenomeSpy [Lavikka et al. 2024] WGBS visualization showing the promoter area of the gene *BRCA1*. While the promoters from GeneHancer (grey) and ENCODE (red) are not capturing the data patterns, the coherently methylated segments found by FUSE (blue and red with shadows) are. (d) The proportion of gained, lost, and preserved breakpoints between FUSE segments under different levels of noise. (e) The F1, precision, sensitivity, and specificity curves of FUSE segment breakpoints with respect to the ground truth.

An overview of the clustering is shown in Fig. 1a. The dissimilarity between two sets of sites A,B⊆{1,…,m} is quantified by the negative log-likelihood ratio (nLLR):


(6) $${\text{nLLR}}_{AB}:=-\text{log}\;{\hat{L}}_{A\cup B}+\text{log}\;{\hat{L}}_{A}+\text{log}\;{\hat{L}}_{B},$$

where each log-likelihood is evaluated at the corresponding MLE ${\hat{p}}_{S,j}$ for each sample. To favor clustering of closely located CpG sites while penalizing long distances, a heavy-tailed genomic distance penalty following the Student’s *t*-distribution with one degree of freedom is introduced.

The complete dissimilarity d(A,B) is defined as


(7) $$\begin{array}{cc} d(A,B)={\text{nLLR}}_{AB} & \\ +\{\begin{array}{cc} -\text{log}\;\left[T\left(\frac{\delta (A,B)}{100};1\right)\right], & \text{if}\;\text{chr}(A)=\text{chr}(B),\\ \infty , & \text{otherwise}. \end{array} & \end{array}$$

Here, $\delta (A,B)=\min_{a\in A,b\in B}|g_{a}-g_{b}|$, where $g_{a}$ is the genomic coordinate of site *a*, denotes the shortest genomic distance between the two sets A and B and T(x;1) is the probability density function of the Student’s *t*-distribution with one degree of freedom.

Clustering is performed agglomeratively producing a hierarchy of segments from fine to coarse resolution. The number of segments can be selected at the desired resolution, with Bayesian Information Criterion (BIC)-based selection (Schwarz 1978) implemented as the default in the R package and used throughout this manuscript. Segmentation is performed independently for each chromosome to ensure scalability to whole-genome WGBS datasets, and results are subsequently merged.

The R-based implementation of the algorithm outputs both a segment-level summary (including genomic coordinates, length, number of CpGs, average methylation across samples) and a segment-by-sample methylation matrix. This preserves within-segment variation across samples and enables downstream analyses, such as differential methylation testing, variability assessment, entropy-based characterization, or stratification by segment properties.

### Coherently methylated segments

Regions with highly coherent methylation patterns are likely actively maintained and might be connected to biological functionality. To test this hypothesis, segments are divided into coherent and non-coherent groups.

To assess the internal coherence of each segment, we test whether all CpG sites in the segment share a common methylation level. The pattern over samples is not constrained and can be heterogeneous. For segment R⊆{1,…,m} containing *r* CpG sites we compare two models:

- Common model $M_{0}$: all sites in *R* share a common methylation rate ${\hat{p}}_{R}$, as given in Eq. 3.
- Separate model $M_{1}$: each site $r_{i}\in R$ has its own methylation rate ${\hat{p}}_{r_{i}}$, as given in Eq. 2.

The corresponding log-likelihoods, $\text{log}\;{\hat{L}}_{R}(M_{0})$ and $\text{log}\;{\hat{L}}_{R}(M_{1})$ are computed using Eq. 5. Under the null hypothesis of a common methylation level, the likelihood ratio test statistic approximately follows a $\chi^{2}$ distribution with r−1 degrees of freedom. Segments with no significant difference between the two models (p>0.05) are considered *coherently methylated*. The coherence-flag is added to the segment-summary output of the R-implementation.

### Code availability

FUSE is implemented as an R package, *methFuse*. The package is available at https://cran.r-project.org/package=methFuse and at https://github.com/holmsusa/methFuse together with examples and documentation.

## Results

### Enrichment of regulatory and repetitive elements among coherently methylated segments

To investigate whether biologically interesting regions co-occur with coherent and likely highly maintained methylation patterns, we evaluated the enrichment of regulatory and repetitive elements within coherently methylated segments. FUSE was applied to 61 WGBS samples from healthy donors obtained from the ENCODE portal (Luo et al. 2020) (Supplementary S1.1, available as supplementary data at *Bioinformatics* online). This resulted in 2931872 segments in chromosomes 1–22, of which 43539 were coherently methylated. Genomic annotations for candidate Cis-Regulatory Elements were retrieved from the ENCODE (ENCSR800VNX), promoter and enhancer regions from GeneHancer (Fishilevich et al. 2017), and repetitive elements from the UCSC Genome Browser RepeatMasker track (Kent et al. 2002) (Smit, Hubley & Green, RepeatMasker Open-4.0. 2013–2015 http://www.repeatmasker.org).

Overlap counts between coherently methylated FUSE segments and each annotation class were determined, and enrichment was assessed using Fisher’s exact test with Benjamini–Hochberg correction. Significant enrichment (FDR < 0.05) was found for three of five ENCODE classes (PLS, pELS, and DNASE_H3K4me3), both GeneHancer categories (promoter and enhancer), and two of twenty-four repetitive element types (retroposon_sva and satellite_telo) (Supplementary S2.1, Table S3, available as supplementary data at *Bioinformatics* online). These results show that coherently methylated FUSE segments overlap known regulatory and repetitive regions.

Analysis of β-value distributions provided a descriptive characterization of the identified segments: as expected by construction, β-value entropy within coherently methylated segments was consistently lower than in the original data across all significantly enriched classes. This reflects the reduced variability that defines coherent segments. Promoter regions from GeneHancer are compared with genome-wide FUSE-segments in Fig. 1b and at the $5^{\prime }$-end of *BRCA1* in Fig. 1c, with analogous plots for other element classes in Figure S3, available as supplementary data at *Bioinformatics* online. An interactive GenomeSpy (Lavikka et al. 2024) visualization of the ENCODE WGBS samples, FUSE-segments, and the functional regions used in this study is available at https://csbi.ltdk.helsinki.fi/p/fuse_encode_gs/.

### Validation of robustness shows high sensitivity in noisy data

To assess the robustness of FUSE against technical variation, we evaluated its ability to recover consistent methylation segments at increasing levels of synthetic noise. Since the segments identified by FUSE are designed to reflect the underlying biological methylation patterns, they are expected to remain largely invariant even when noise is introduced.

A baseline, noise-free dataset $D_{0}=\{C_{0},T_{0}\}$ and a collection of noisy data sets


(8) $$D_{\eta }=\{D_{\eta }^{\left(k\right)}=\{C_{\eta }^{\left(k\right)},T_{\eta }^{\left(k\right)}\}|k=1,2,\ldots ,100\}$$

of noise amplitudes η={5,10,…,120} % were constructed as described in Supplementary 3.1, available as supplementary data at *Bioinformatics* online.

FUSE was independently applied to each $D_{\eta }^{\left(k\right)}\in D_{\eta }$ across all noise levels, and the resulting segmentation was compared to the ground truth segments in $D_{0}$ to identify preserved, lost, and gained breakpoints. For each noise level, the relative proportions of these breakpoint categories were summarized in Fig. 1d. Performance metrics including sensitivity, specificity, precision, and the F1 score were computed, and their 95% confidence intervals were estimated using bootstrapping, see Fig. 1e.

FUSE maintained high sensitivity in detecting true breakpoints across all noise levels (Fig. 1d), indicating that the underlying methylation pattern could be recovered even under substantial noise. As the noise increased to over 100%, a spike in false positive breakpoints was observed (Fig. 1d). In a setting where the noise is 100% or more, most CpG sites have more noise than signal, and the segmentation becomes completely random. At noise levels between 60% and 100% there are already more gained breakpoints than original ones, meaning some over-segmentation of contiguous regions is happening. In practical applications, the hierarchical structure of FUSE allows exploration of multiple resolutions: segments can be analyzed individually or aggregated into broader regions for downstream biological analyses, ensuring flexibility in assessing regulatory domains of varying size.

### Performance evaluation

Computational benchmarking on the 61-sample ENCODE WGBS dataset demonstrated that full-genome segmentation can be completed in approximately 61 minutes with peak memory usage of 15 GB when processed chromosome-wise (Supplementary S1.2, available as supplementary data at *Bioinformatics* online).

We compared FUSE against established tools [Bumphunter (Jaffe et al. 2012, Aryee et al. 2014), DSS (Wu et al. 2013, Feng et al. 2014, Wu et al. 2015, Park and Wu 2016), MethSeg (Akalin et al. 2012)] and a fixed-window baseline in terms of runtime, memory usage, and segmentation accuracy (Supplementary S3.2, available as supplementary data at *Bioinformatics* online). FUSE achieved the highest sensitivity in breakpoint recovery, while runtime and memory usage were comparable across tools.

## Conclusions

In this study, we introduced FUSE, a data-driven segmentation framework for capturing spatially homogeneous methylation blocks in WGBS data. FUSE derives methylation segments over the sites directly from the observed DNAm pattern while allowing different methylation states over samples, providing a cohort-specific representation of the underlying co-methylated structure. The hierarchical clustering approach allows the recovery of segments at any resolution, enabling flexible exploration of methylation patterns across scales. As with any data-driven consensus segmentation, the resulting segment boundaries reflect the biological context of the given study population. If sample-specific boundaries are of interest, FUSE can be applied to individual samples or subsets of samples to enable direct comparison of segment architectures across conditions.

We showed that segments with coherent methylation patterns, likely reflecting highly maintained methylation, coincide with known regulatory and repeated regions, which can be further investigated in the provided GenomeSpy visualization.

FUSE is implemented as an easy-to-use R package, *methFuse*, with optimized C-based core functions for computational efficiency. Beyond segmentation, FUSE can serve as a foundation for downstream applications, such as differential methylation analysis, integration with chromatin-state maps, or epigenetic biomarker discovery.

Future work may add more focus on the heterogeneous segments and characterize their biological characteristics. Another direction would be to extend FUSE to multi-omics contexts, allowing segmentation informed by both methylation and chromatin accessibility data, or to non-CpG methylation patterns in diverse organisms. Overall, FUSE provides a robust and interpretable framework for uncovering biologically meaningful methylation domains, advancing our ability to study epigenetic regulation at the genome scale.

## Supplementary Material

btag201_Supplementary_Data
