# Supplementary material for: FUSE: data-driven functional segmentation of DNA methylation data
Source: Bioinformatics. 2026 Apr 29;42(5):btag201. doi: 10.1093/bioinformatics/btag201 (PMC13181182; doi:10.1093/bioinformatics/btag201)
Supplement: btag201_Supplementary_Data [file btag201_supplementary_data.pdf]

FUSE supplementary material

S1. Running FUSE on ENCODE dataset

S1.1 ENCODE data set

In this paper, FUSE was applied to a whole-genome bisulfite sequencing (WGBS) dataset consisting of 61 healthy donor samples (Supplementary Table 1) obtained from the ENCODE portal (Luo et al., 2019). Everything but chromosomes 1-22 and X were deleted, and the samples were merged into two tables, methylated counts (C) and unmethylated counts (T).

Before applying FUSE, every CpG site with missing values in more than 50% of the samples was removed.

Supplementary table 1: ENCODE WGBS samples used in this paper

sample	patient	tissue	gender	age
ENCFF477GKI	ENCDO424HVB	adipose tissue	F	30
ENCFF210XTE	ENCDO424HVB	adrenal gland	F	30
ENCFF913ZNZ	ENCDO793LXB	adrenal gland	F	53
ENCFF553HJV	ENCDO424HVB	aorta	F	30
ENCFF076CIO	ENCDO793LXB	esophagus squamous epithelium	F	53
ENCFF510EMT	ENCDO424HVB	esophagus	F	30
ENCFF577TCU	ENCDO793LXB	gastroesophageal sphincter	F	53
ENCFF121VIX	ENCDO271OUW	lower leg skin	F	51
ENCFF039JFT	ENCDO424HVB	lung	F	30
ENCFF247ILV	ENCDO424HVB	ovary	F	30
ENCFF189WPY	ENCDO271OUW	ovary	F	51
ENCFF454NPL	ENCDO271OUW	ovary	F	51
ENCFF303ZGP	ENCDO793LXB	ovary	F	53
ENCFF716SXG	ENCDO793LXB	ovary	F	53
ENCFF500DKA	ENCDO424HVB	pancreas	F	30
ENCFF913UZU	ENCDO424HVB	psoas muscle	F	30
ENCFF577VGR	ENCDO793LXB	right lobe of liver	F	53
ENCFF266NGW	ENCDO424HVB	small intestine	F	30
ENCFF730NQT	ENCDO424HVB	spleen	F	30
ENCFF509SPS	ENCDO271OUW	spleen	F	51
ENCFF550FZT	ENCDO271OUW	spleen	F	51
ENCFF435SPL	ENCDO424HVB	stomach	F	30
ENCFF811QOG	ENCDO271OUW	stomach	F	51

ENCFF497IYX	ENCDO271OUW	thyroid gland	F	51
ENCFF843SYR	ENCDO793LXB	tibial nerve	F	53
ENCFF733EFJ	ENCDO271OUW	upper lobe of left lung	F	51
ENCFF588IUK	ENCDO222AAA	muscle	F	0
ENCFF672QKY	ENCDO222AAA	muscle	F	0
ENCFF699GKH	ENCDO830BUW	mammary	F	50
ENCFF874GGB	ENCDO830BUW	mammary	F	50
ENCFF318AMC	ENCDO058AAA	adipose tissue	M	34
ENCFF216DJL	ENCDO058AAA	adrenal gland	M	34
ENCFF618WAT	ENCDO451RUA	adrenal gland	M	54
ENCFF699RBP	ENCDO845WKR	body of pancreas	M	37
ENCFF748MTS	ENCDO451RUA	body of pancreas	M	54
ENCFF952NTB	ENCDO451RUA	esophagus squamous epithelium	M	54
ENCFF625GVK	ENCDO058AAA	esophagus	M	34
ENCFF237MNK	ENCDO451RUA	gastroesophageal sphincte	M	54
ENCFF536RSX	ENCDO058AAA	heart left ventricle	M	34
ENCFF513ITC	ENCDO058AAA	heart right ventricle	M	34
ENCFF219GCQ	ENCDO845WKR	lower leg skin	M	37
ENCFF763RUE	ENCDO058AAA	pancreas	M	34
ENCFF027KTR	ENCDO451RUA	prostate gland	M	54
ENCFF110AZO	ENCDO058AAA	right cardiac atrium	M	34
ENCFF455TQO	ENCDO058AAA	sigmoid colon	M	34
ENCFF122LEF	ENCDO058AAA	small intestine	M	34
ENCFF526PFA	ENCDO058AAA	spleen	M	34
ENCFF333OHK	ENCDO845WKR	spleen	M	37
ENCFF956ACC	ENCDO845WKR	spleen	M	37
ENCFF497YOO	ENCDO058AAA	stomach	M	34
ENCFF844EFX	ENCDO451RUA	stomach	M	54
ENCFF638QVP	ENCDO845WKR	testis	M	37
ENCFF715DMX	ENCDO451RUA	testis	M	54
ENCFF223LJW	ENCDO845WKR	thyroid gland	M	37
ENCFF699KTW	ENCDO451RUA	tibial nerve	M	54
ENCFF424XKF	ENCDO451RUA	transverse colon	M	54
ENCFF842MHJ	ENCDO845WKR	upper lobe of left lung	M	37
ENCFF435ETE	ENCDO661BYS	nk	M	37
ENCFF689TNG	ENCDO661BYS	mono	M	37
ENCFF703XLD	ENCDO661BYS	bcell	M	37
ENCFF953DKC	ENCDO661BYS	tcell	M	37

S1.2 Computational benchmarking

For the computational benchmarking, FUSE was applied on the 61 ENCODE samples described in S1.1. Input data were preprocessed into chromosome-wise matrices (CpGs × samples), and each chromosome was processed independently. Runtime, peak memory usage, and CPU utilization were recorded for each chromosome and are reported in Supplementary Table 2. When executed sequentially across chromosomes, the total runtime was approximately 61 minutes, with per-chromosome runtimes ranging from ~1 to 4 minutes. Peak memory usage was approximately 15 GB. All benchmarks were performed on a Linux-based SLURM-managed high-performance computing cluster using a single compute node with 56 CPU cores and 355 GB RAM. Segmentation of each chromosome was performed using a single core.

Supplementary Table 2: Runtime, peak memory usage, CPU utilization, and resulting number of segments when applying FUSE to the ENCODE WGBS dataset, reported per chromosome.

Chromosome	Runtime (min:sec)	MaxRAM (KB)	CPU (%)	Number of segments
chr1	4:01.08	14956092	99 %	238339
chr2	3:53.44	14983468	91 %	238746
chr3	3:15.82	11315300	91 %	193821
chr4	2:59.14	10808248	90 %	190475
chr5	3:59.32	11166380	94 %	180163
chr6	3:59.50	10425468	94 %	176751
chr7	4:06.47	12093960	94 %	170973
chr8	3:42.01	9515196	94 %	154629
chr9	3:04.45	8546824	94 %	121960
chr10	2:38.44	9872944	91 %	150331
chr11	2:29.69	9672080	92 %	146457
chr12	3:15.50	9393840	94 %	147175
chr13	2:15.48	6219936	94 %	112170
chr14	1:34.92	6331128	92 %	98221
chr15	1:36.23	5760924	90 %	88182
chr16	2:35.91	6885552	94 %	98634
chr17	2:47.48	8362072	95 %	95744
chr18	1:51.62	5665804	93 %	86791
chr19	1:51.32	7149676	91 %	73492

chr20	1:25.56	5739180	91 %	75181
chr21	0:58.84	3037160	93 %	47545
chr22	1:30.21	4075508	94 %	46092
chrX	1:56.19	8387684	97 %	97884

S2. Biological validation

Applying FUSE on the ENCODE data set resulted in 3 029 756 segments in chromosomes 1 – 22 and X. To demonstrate that this data-driven segmentation captures biologically relevant patterns in the genome, the segments were compared to the following biologically relevant and annotated regions: the candidate Cis-Regulatory Elements from the ENCODE database (Luo et al., 2019), accession ID ENCSR800VNX, promoters and enhancers from the GeneHancer database (Fishilevich et al., 2017), and the repeated elements from the RepeatMasker annotation track in the UCSC Genome Browser (Kent et al., 2002, Smit et al.).

S2.1 Alignment of FUSE segments with regulatory and repeated regions

To test the biological relevance of the global segmentation, the alignment of segment boundaries and functional region boundaries were assessed. Here all regions from the ENCODE cis-regulatory element database, GeneHancer promoter and enhancer regions, as well as all repeated regions from the RepeatMasker track in the UCSC genome browser were used. For all elements belonging to the same type of region, the distances from the closest lying segment boundaries to the start and end points of the elements were measured, and the mean and median boundary distances were computed. To show that the biological regions aligned better with FUSE segments than with any random segmentation, a bootstrap test with 100 bootstraps was performed. For each bootstrap round, a number *k* between 0 and the number of CpG sites in the genome was randomly drawn. The FUSE segments were then shifted forward *k* steps, and the mean and median boundary distances were computed. The shifting approach ensured that the length distribution of the segments stayed comparable to the original FUSE segmentation. A one-sided t-test was used for testing significance. The results in Supplementary Figure 1 show that for all regions except *satellite_telo, satellite_acro*, *retroposon_sva*, *DNA_PIF-Harbinger*, and *PLS*, the median boundary distance between FUSE segments and biological regions is smaller than random. In Supplementary Figure 2, it is shown that for all regions except *scRNA_scRNA*, *satellite_telo*, *satellite_satellite*, *satellite_centro*, *satellite_acro,* and *Promoter*, the mean boundary distance between FUSE segments and regions is better than random. These

findings indicate that the data-driven FUSE segmentation captures biologically relevant patterns globally.

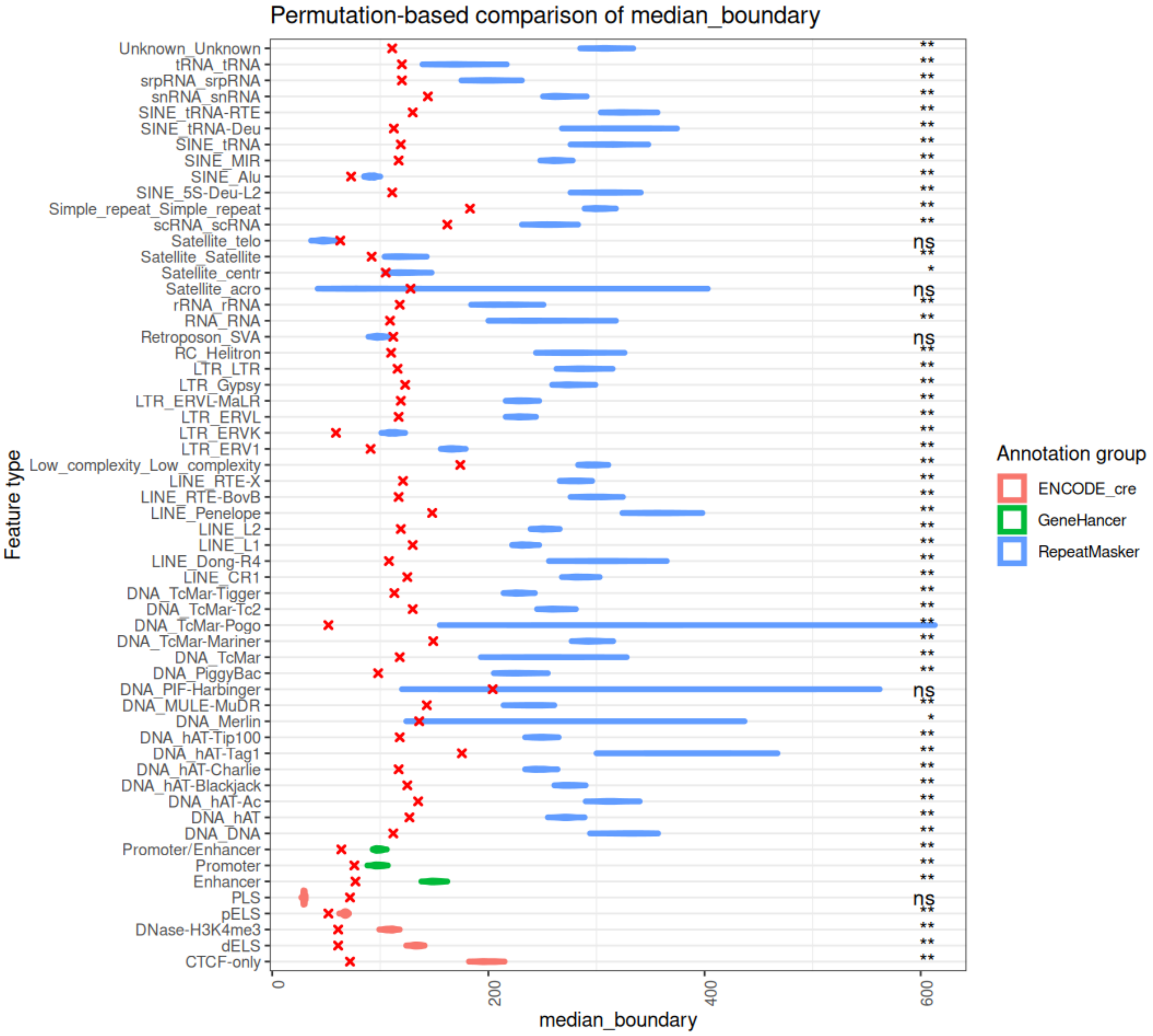

Supplementary Figure 1: Median boundary distance between regions and segments. The range of results from the bootstraps are plotted as blue bars, and the results from the original FUSE segmentation are plotted as red crosses.

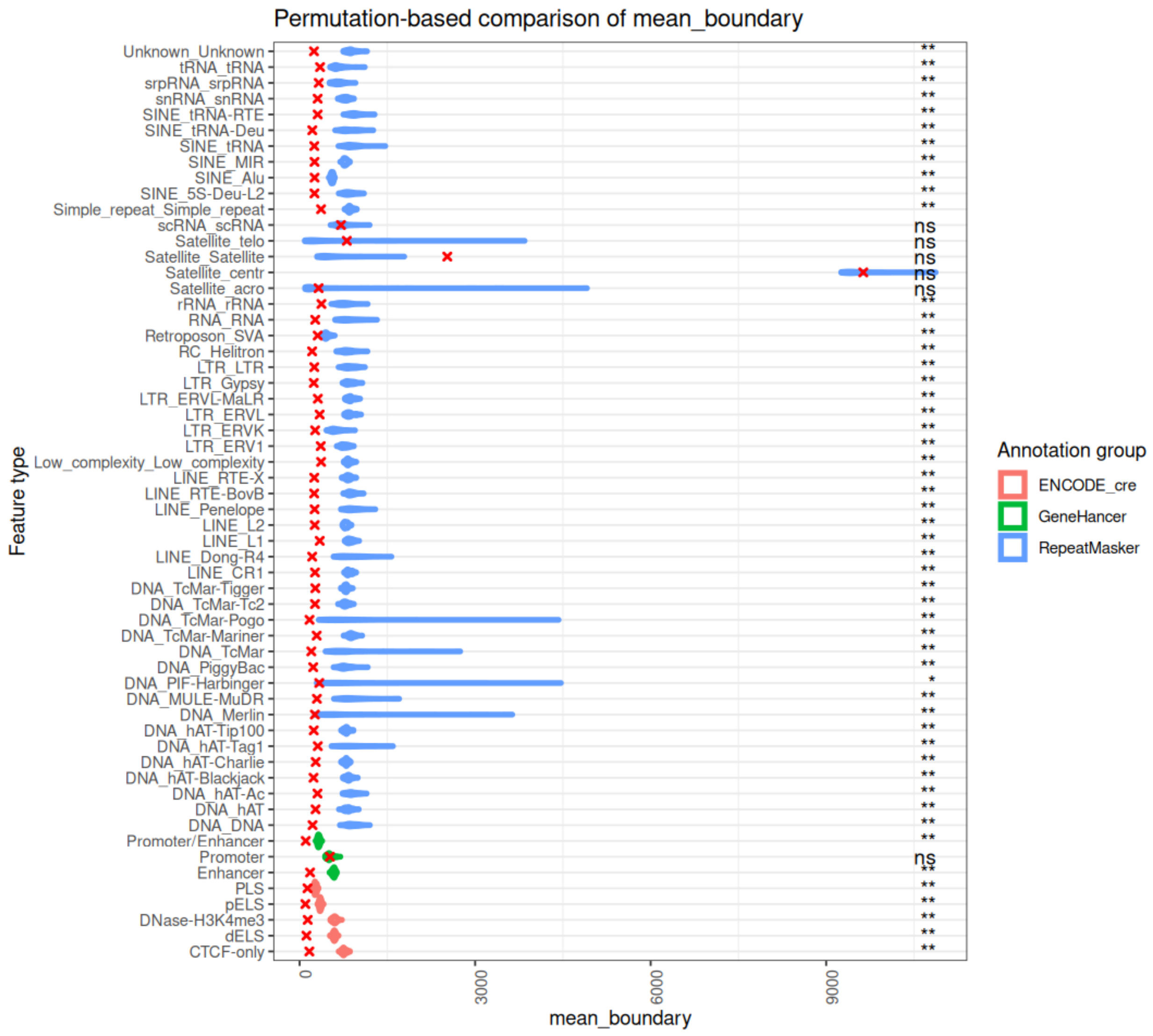

Supplementary Figure 2: Mean boundary distance between regions and segments. The range of results from the bootstraps are plotted as blue bars, and the results from the original FUSE segmentation are plotted as red crosses.

S2.1 Coherent segments overlap with biologically relevant regions

Of the 2 931 872 segments in chromosomes 1-22, there were 43 539 coherently methylated ones with a length of at least 4 CpGs. To show that coherent segments overlap biologically relevant regions more often than other segments, overlap between coherent segments and the biological regions were computed. All regions from the ENHANCER Cis-Regulatory Elements table were used, as well as all promoters and enhancers from GeneHancer. From the repeated regions, all the region families occurring at least 1000 times in the genome

were selected, to ensure reliable global results. A segment is considered to overlap a biological region if any part of the segment and region overlaps.

The enrichment of biological region types among the coherently methylated segments was assessed using Fisher's test, and the results are shown in Supplementary Table 3. Seven of the 31 region types were significantly enriched among the coherently methylated segments, including *DNASE-H3K4me3*, *pELS*, and *PLS* from ENCODE, *promoters* and *enhancers* from GeneHancer, and *retroposon_sva* and *satellite_telo* from RepeatMasker.

Supplementary Table 3: Result of Fisher's test for enrichment of regions among coherently methylated segments.

Data source	Region type	Odds_Ratio	FDR_BH	Significant
ENCODE	PLS	74.88278	0.00E+00	TRUE
RepeatMasker	Retroposon_SVA	21.462001	0.00E+00	TRUE
ENCODE	pELS	19.562791	0.00E+00	TRUE
GeneHancer	Promoter	19.111964	0.00E+00	TRUE
RepeatMasker	Satellite_telo	4.046539	6.75E-16	TRUE
GeneHancer	Enhancer	3.84705	0.00E+00	TRUE
ENCODE	DNase-H3K4me3	2.174514	1.59E-100	TRUE
RepeatMasker	Satellite_Satellite	0.8266	1.95E-01	FALSE
RepeatMasker	LTR_ERV1	0.721989	3.00E-47	TRUE
ENCODE	dELS	0.565076	0.00E+00	TRUE
ENCODE	CTCF-only	0.453368	1.64E-49	TRUE
RepeatMasker	SINE_5S-Deu-L2	0.422992	4.55E-04	TRUE
RepeatMasker	SINE_MIR	0.400727	0.00E+00	TRUE
RepeatMasker	SINE_Alu	0.353554	0.00E+00	TRUE
RepeatMasker	LTR_ERVK	0.346434	2.40E-35	TRUE
RepeatMasker	Satellite_centr	0.29863	4.94E-82	TRUE
RepeatMasker	LINE_Penelope	0.296775	5.86E-03	TRUE
RepeatMasker	LINE_L2	0.279878	0.00E+00	TRUE
RepeatMasker	LINE_RTE-BovB	0.259443	2.79E-21	TRUE
RepeatMasker	SINE_tRNA-Deu	0.245859	3.19E-02	TRUE
RepeatMasker	LINE_CR1	0.203791	1.70E-167	TRUE
RepeatMasker	SINE_tRNA	0.195055	2.28E-07	TRUE
RepeatMasker	LINE_L1	0.169323	0.00E+00	TRUE
RepeatMasker	LTR_ERVL	0.139166	0.00E+00	TRUE
RepeatMasker	LTR_Gypsy	0.13681	6.09E-55	TRUE
RepeatMasker	SINE_tRNA-RTE	0.126365	7.30E-20	TRUE

RepeatMasker	LTR_ERVL-MaLR	0.102516	0.00E+00	TRUE
RepeatMasker	LINE_RTE-X	0.08658	3.02E-60	TRUE
RepeatMasker	LTR_LTR	0.024893	6.18E-16	TRUE
RepeatMasker	LINE_Dong-R4	0	1.95E-03	TRUE
RepeatMasker	Satellite_acro	0	4.14E-01	FALSE

By construction, FUSE segments have a lower entropy than random segments. The entropies over CpG sites for the biological regions enriched among coherent FUSE segments were computed, together with the entropies of all FUSE-segments overlapping the regions and coherently labeled FUSE-segments overlapping the regions. The β-value distributions and entropy values in these three cases are shown in Supplementary Figure 3.

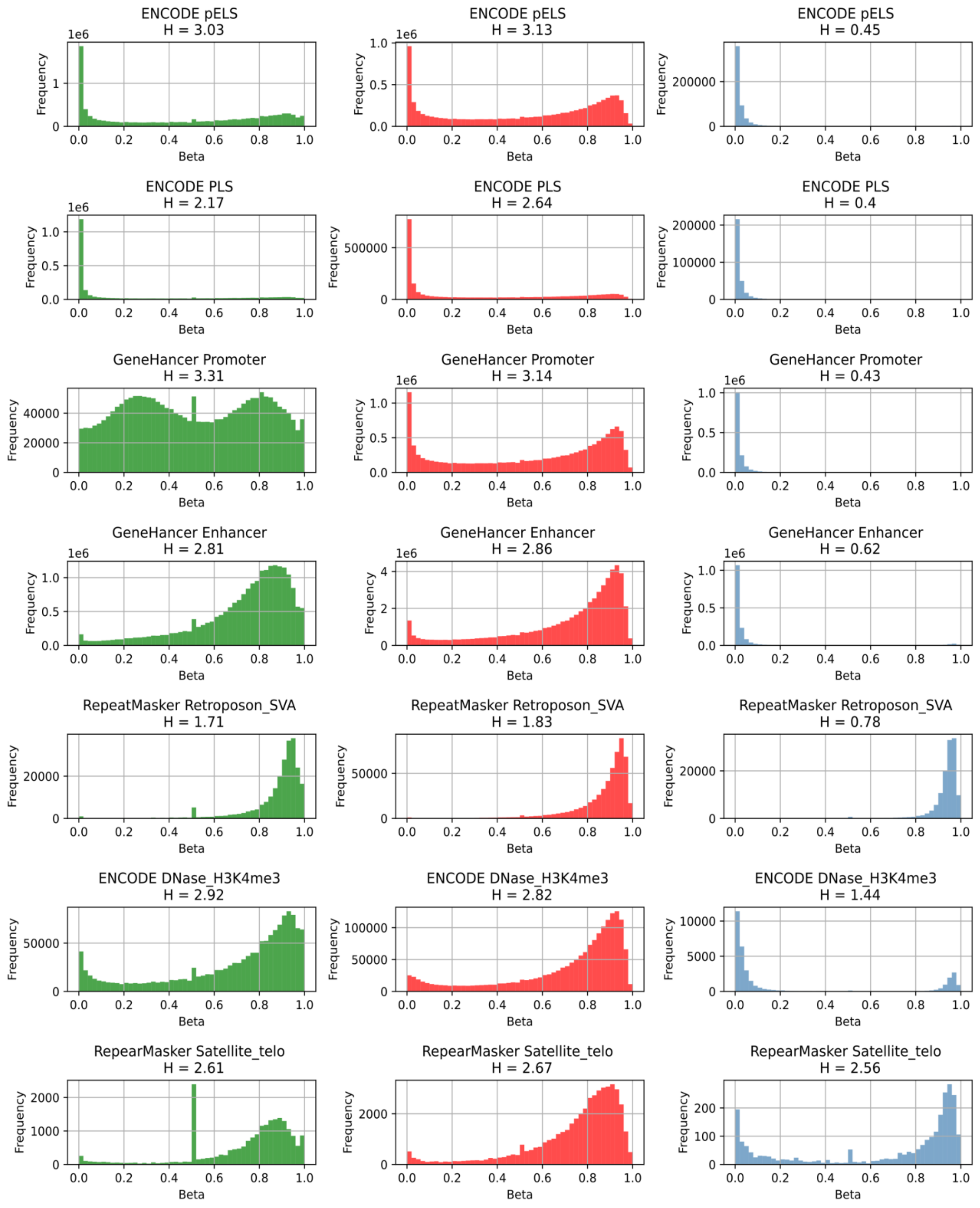

Supplementary Figure 3: Comparison of β distributions in the seven enriched regions. In the left column are the regions as they are annotated, in the center column are all FUSE-segments overlapping with these regions, and in the right column are all coherently

methylated FUSE segments overlapping with these regions. All regions show a decrease in entropy (H) of the β-values when using the FUSE coherently methylated segments instead of the original annotated regions.

S3. Technical validation

S3.1 Testing robustness against noise using simulated data

To assess the robustness of FUSE against technical variation, we evaluated its ability to recover consistent methylation segments at increasing levels of synthetic noise. Since the segments identified by FUSE are designed to reflect underlying biological methylation patterns, they are expected to remain largely invariant even when noise is introduced. A baseline, noise-free dataset $D_0 = \{C_0, T_0\}$ was constructed to serve as ground truth. Starting from the ENCODE WGBS data set described above, we restricted the analysis to chromosome 20 to ensure computational feasibility. All 61 samples were summed to represent one ”global” methylome. For each of the 1 546 954 CpG sites, β-values were computed and discretized into two methylation states: fully unmethylated (β ≤ 0.3) and fully methylated (β ≥ 0.7). Sites with intermediate methylation (0.3 < β < 0.7) were assigned the state of their immediate 5' neighbor to preserve local continuity. Both cytosines within each CpG dinucleotide were constrained to share the same state. This procedure produced a binary methylation map with well-defined segment boundaries, used as the ground truth segmentation G_0. The count matrices $C_0, T_0 \in \mathbb{R}^{1546954 \times 20}$ were constructed from G_0 as follows: C_0 contains 20 replicates of G_0 multiplied by the read depth 30x representing methylated counts. T_0 contains 20 replicates of $1 - G_0$ multiplied by 30x representing unmethylated counts.

To generate noisy data sets, synthetic perturbations of amplitude $\eta = \{5, 10, \ldots, 120\}$ relative to the sequencing depth 30 × were added to C_0 and T_0. For each CpG site *i*, 40 random values were drawn from a uniform distribution in $[-(30x \times \eta), (30x \times \eta)]$; the first 20 were added to site *i* in C_0, and the rest to site *i* in T_0. Negative counts were truncated to zero. For each noise level η, 100 independent replicates

$$D_\eta = \left\{D_\eta^{(k)} = \left\{C_\eta^{(k)}, T_\eta^{(k)}\right\} | k = 1,2, \ldots, 100| \right\}$$

were generated.

S3.2 Benchmarking against other tools

Three tools were selected for benchmarking: Bumphunter (version 1.52.0) (Jaffe et al. 2012, Aryee et al. 2014), to highlight a regional smoothing baseline, DSS (version 2.58.0) (Wu et al. 2013, Feng et al. 2014, Wu et al. 2015, Park et al. 2016) as a statistical DMR caller, and MethSeg (from package methylKit version 1.36.0) (Akalin et al. 2012) as a global segmentator. A fixed-window approach with windows of size 1000 was included to show a non-adaptive baseline.

S3.2.1 Benchmarking outline

The performance of FUSE compared to other segmentation tools and DMR callers under increasing levels of noise was first assessed on the simulated data set D_0 created in S3.1, but on 4 identical replicates instead of 20. Since this data was constructed specifically for demonstrating the performance of FUSE, it does not contain DMRs. To make a fairer comparison for the DMR callers, another data set, including DMRs, was constructed in the following way: 4 replicates were selected from D_0, and two of them (group B) were flipped to the have the exact opposite methylation pattern compared to the first two (group A). This created a dataset with perfect DMRs between the two groups everywhere. To make it more natural, 20% of randomly chosen DMRs were flipped back, so that the two groups had identical methylation. This created 4 types of segments: 1) DMR where group A is hypermethylated and B is hypomethylated (40 % of segments), 2) DMR where group A is hypomethylated and B is hypermethylated (40 % of segments), 3) non-DMR where both groups are hypomethylated (10 %) and 4) non-DMR where both groups are hypermethylated (10 %).

S3.2.2 Test A: recovering segments in identical replicates

All 4 tools and the fixed window approach were applied to the simulated data set with 4 identical replicates, and the number of lost, gained and preserved breakpoints were recorded for each noise level like in S3.1. The F1-metric, precision, sensitivity and specificity, including their confidence intervals computed through parametric multinomial bootstrap of the confusion matrix, were computed for each tool and noise level. The results in Supplementary Figure 4 show that while FUSE has the highest sensitivity, its specificity decreases rapidly around a noise level of 1.0 (100% added noise). This is expected, as the areas with a similar methylation pattern get shattered when more noise than signal is present. MethSeg showed strong robustness to increasing noise levels and consistently high precision. DSS did not find any segments in this setting at any noise level, which is expected by a DMR caller.

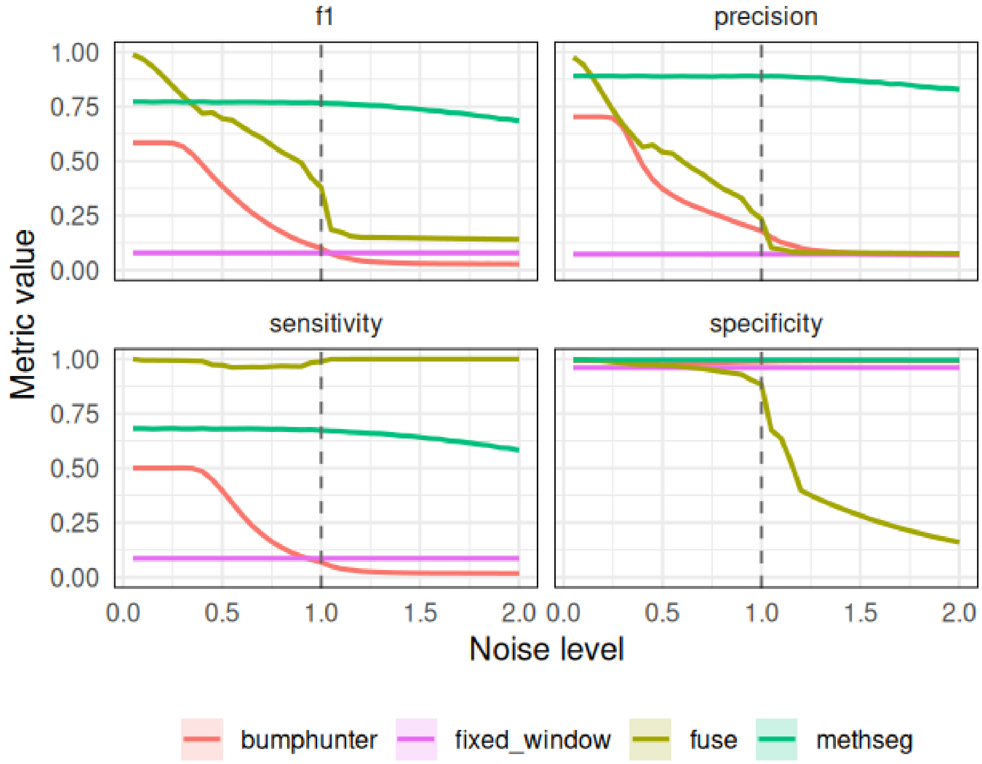

Supplementary Figure 4: The segmentation performance of FUSE, Bumphunter, MethSeg and the fixed window baseline on 4 identical replicates across different noise levels. Results for DSS are not included since DSS didn't find any regions at all in this setting.

S3.2.3 Test B: recovering segments and DMRs in presence of DMRs

All four tools and the fixed windows approach were applied on the four replicates consisting of groups A and B as described above, across the same noise levels as previously. First, the ability to find all the segments, both DMRs and non-DMRs, was tested. Supplementary Figure 5 shows that, again, FUSE shows decreasing specificity when noise levels exceed 1.0, consistent with over-segmentation under extreme noise conditions, but has best overall sensitivity. Again, MethSeg showed strong robustness to increasing noise levels and consistently high precision. This time DSS found segments up to a noise level of 1.3, but after that none.

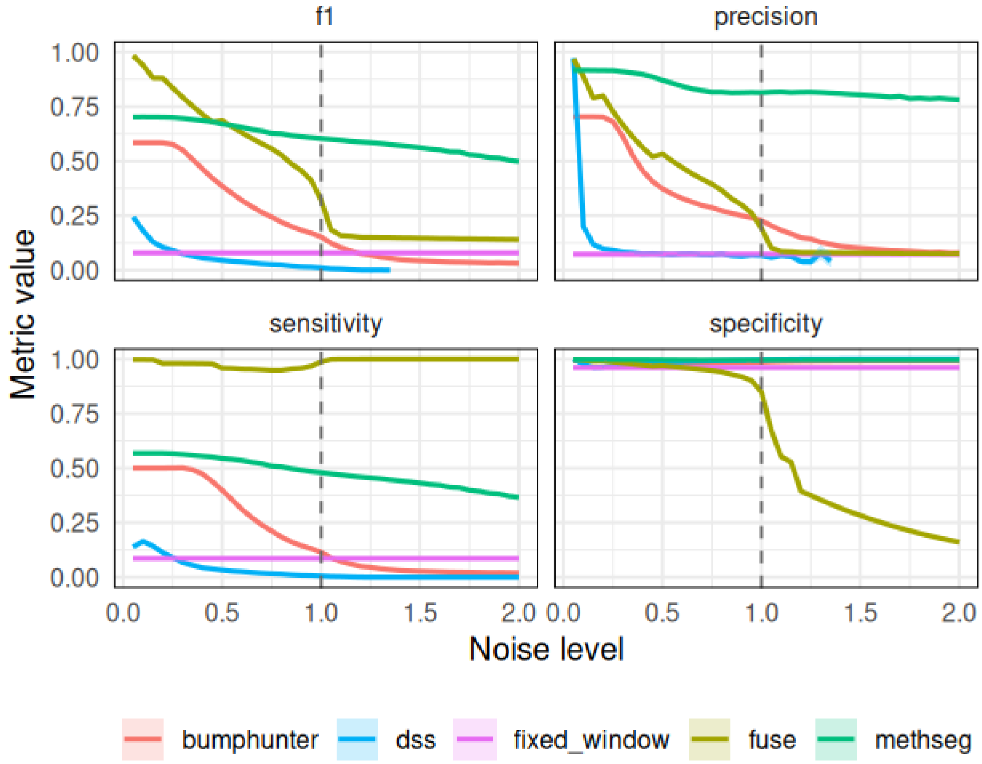

Supplementary Figure 5: The segmentation performance of FUSE, Bumphunter, DSS, MethSeg, and the fixed window baseline on four grouped replicates across different noise levels.

Next, the segments discovered by the three DMR-callers, Bumphunter, DSS, and MethSeg, were compared against only DMR segments. The results in Supplementary Figure 6 show that MethSeg recovers DMRs the best and is the most invariant to noise, while DSS performs the worst.

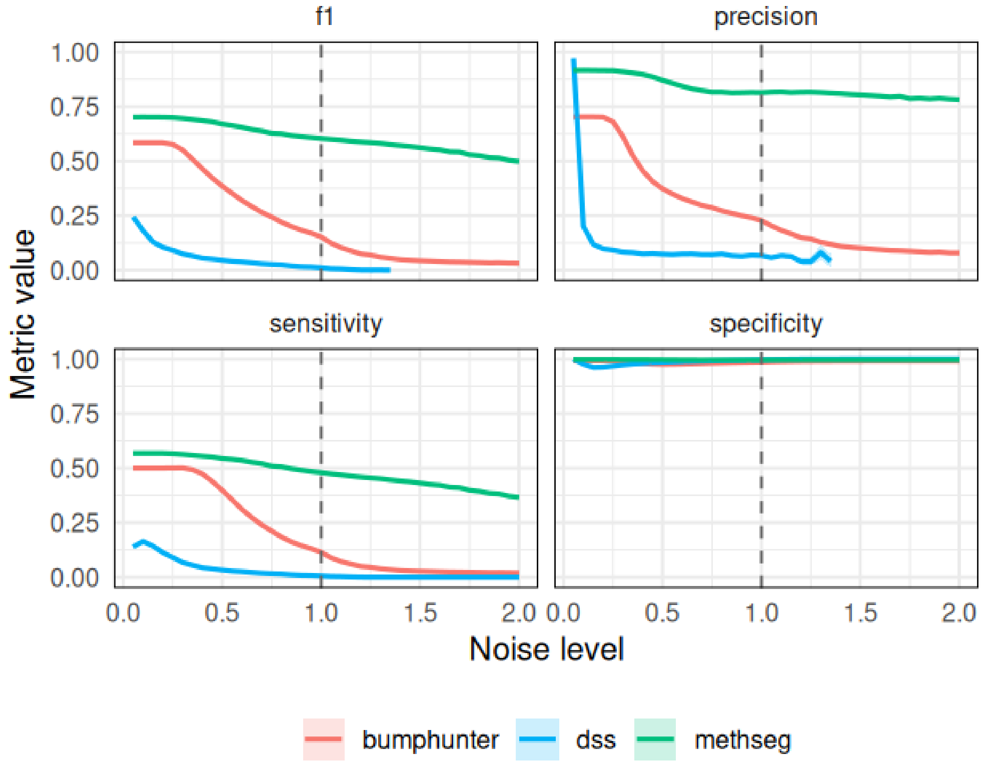

Supplementary Figure 6: The DMR-calling performance of Bumphunter, DSS, and MethSeg on four grouped replicates across different noise levels.

S3.2.4 Benchmarking runtimes and memory usage

The four tools and the fixed window baseline were applied on 4 samples from the ENCODE data set, accession IDs ENCFF110AZO, ENCFF216DJL, ENCFF913UZU, and ENCFF303ZGP. The former two are from males, and the latter two from females, and these were the groupings used for the tools needing groups (DSS, Methseg, and Bumphunter). The tools were run on each chromosome separately, and the performance was recorded with the bash command `/usr/bin/time -v -o`. The benchmarking was performed on an Ubuntu system using slurm, and each job was assigned 8 CPUs. However, only DSS was able to perform parallel computing per chromosome.

The results from the benchmarking in Supplementary Figure 7 show that Bumphunter is an outlier in terms of runtime. Running on chromosome 1 took over 1h for Bumphunter, and only chromosome 21 took less than 5 minutes. All the other tools finished running on each chromosome in less than 5 minutes, except for DSS on chromosome 1 which took slightly longer than 5 minutes. Max RSS are quite similar for all tools, ranging from 5 GB to 6 GB for

the largest chromosome 1, and between 1 GB and 2 GB for the smallest chromosome 21. All tools except DSS used around 100 % CPU, while DSS used between 380 – 530 % CPU for each chromosome.

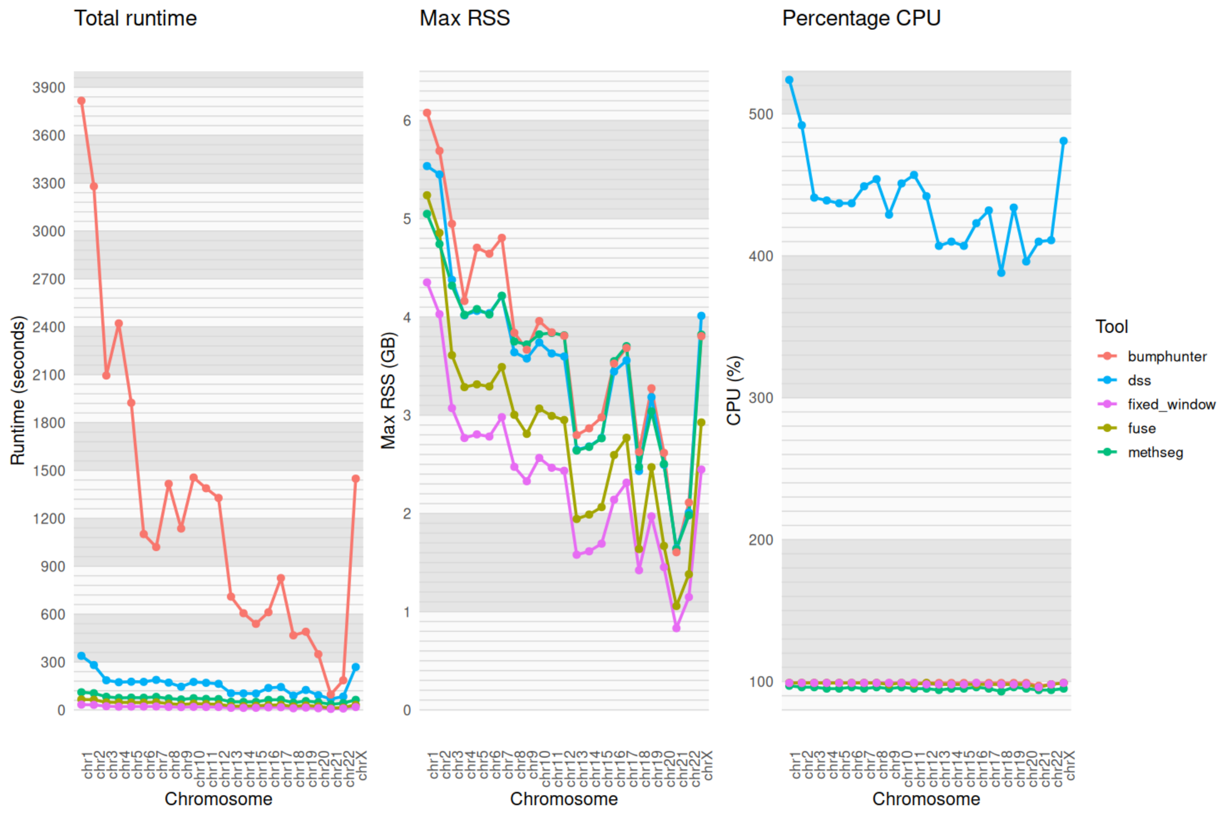

Supplementary Figure 7: Total runtime, max RSS, and percentage CPU per chromosome for the tested tools.

References

Altuna Akalin, Matthias Kormaksson, Sheng Li, Francine E Garrett-Bakelman, Maria E Figueroa, Ari Melnick and Christopher E Mason. methylKit: a comprehensive R package for the analysis of genome-wide DNA methylation profiles. Genome Biology 13:R87 (2012).

Martin J. Aryee, Andrew E. Jaffe, Hector Corrada-Bravo, Christine Ladd-Acosta, Andrew P. Feinberg, Kasper D. Hansen, Rafael A. Irizarry, Minfi: a flexible and comprehensive Bioconductor package for the analysis of Infinium DNA methylation microarrays,

Bioinformatics, Volume 30, Issue 10, May 2014, Pages 1363–1369, https://doi.org/10.1093/bioinformatics/btu049

Hao Feng, Karen N. Conneely, Hao Wu, A Bayesian hierarchical model to detect differentially methylated loci from single nucleotide resolution sequencing data, *Nucleic Acids Research*, Volume 42, Issue 8, 1 April 2014, Page e69, https://doi.org/10.1093/nar/gku154

S. Fishilevich, R. Nudel, N. Rappaport, R. Hadar, I. Plaschkes, T. Iny Stein, N. Rosen, A. Kohn, M. Twik, M. Safran, D. Lancet, and D. Cohen. Genehancer: genome-wide integration of enhancers and target genes in genecards. Database, 2017, Jan. 2017. ISSN 1758-0463. doi: 10.1093/database/bax028. URL http://dx.doi.org/10.1093/database/bax028.

Andrew E Jaffe, Peter Murakami, Hwajin Lee, Jeffrey T Leek, M Daniele Fallin, Andrew P Feinberg, Rafael A Irizarry, Bump hunting to identify differentially methylated regions in epigenetic epidemiology studies, International Journal of Epidemiology, Volume 41, Issue 1, February 2012, Pages 200–209, https://doi.org/10.1093/ije/dyr238

W. J. Kent, C. W. Sugnet, T. S. Furey, K. M. Roskin, T. H. Pringle, A. M. Zahler, and D. Haussler. The human genome browser at ucsc. Genome Research, 12(6):996–1006, 2002. doi: 10.1101/gr.229102.

Y. Luo, B. C. Hitz, I. Gabdank, J. A. Hilton, M. S. Kagda, B. Lam, Z. Myers, P. Sud, J. Jou, K. Lin, U. K. Baymuradov, K. Graham, C. Litton, S. R. Miyasato, J. S. Strattan, O. Jolanki, J.-W. Lee, F. Y. Tanaka, P. Adenekan, E. O'Neill, and J. M. Cherry. New developments on the encyclopedia of dna elements (encode) data portal. Nucleic Acids Research, 48(D1):D882–D889, Nov. 2019. ISSN 1362-4962. doi: 10.1093/nar/gkz1062. URL http://dx.doi.org/10.1093/nar/gkz1062.

Yongseok Park, Hao Wu, Differential methylation analysis for BS-seq data under general experimental design, *Bioinformatics*, Volume 32, Issue 10, May 2016, Pages 1446–1453, https://doi.org/10.1093/bioinformatics/btw026

A. F. A. Smit, R. Hubley, and P. Green. Repeatmasker open-4.0. http://www.repeatmasker.org. Accessed September 12, 2025

Hao Wu, Chi Wang, Zhijin Wu, A new shrinkage estimator for dispersion improves differential expression detection in RNA-seq data, *Biostatistics*, Volume 14, Issue 2, April 2013, Pages 232–243, https://doi.org/10.1093/biostatistics/kxs033

Hao Wu, Tianlei Xu, Hao Feng, Li Chen, Ben Li, Bing Yao, Zhaohui Qin, Peng Jin, Karen N. Conneely, Detection of differentially methylated regions from whole-genome bisulfite

sequencing data without replicates, *Nucleic Acids Research*, Volume 43, Issue 21, 2 December 2015, Page e141, https://doi.org/10.1093/nar/gkv715
